# Genotyping for Human Papillomavirus (HPV) 16/18/52/58 Has a Higher Performance than HPV16/18 Genotyping in Triaging Women with Positive High-risk HPV Test in Northern Thailand

**DOI:** 10.1371/journal.pone.0158184

**Published:** 2016-06-23

**Authors:** Surapan Khunamornpong, Jongkolnee Settakorn, Kornkanok Sukpan, Prapaporn Suprasert, Jatupol Srisomboon, Suthida Intaraphet, Sumalee Siriaunkgul

**Affiliations:** 1 Department of Pathology, Faculty of Medicine, Chiang Mai University, Chiang Mai, Thailand; 2 Department of Obstetrics and Gynecology, Faculty of Medicine, Chiang Mai University, Chiang Mai, Thailand; 3 Boromarajonani College of Nursing, Khon Kaen, Thailand; Georgetown University, UNITED STATES

## Abstract

**Background:**

Testing for high-risk human papillomavirus DNA (HPV test) has gained increasing acceptance as an alternative method to cytology in cervical cancer screening. Compared to cytology, HPV test has a higher sensitivity for the detection of histologic high-grade squamous intraepithelial lesion or worse (HSIL+), but this could lead to a large colposcopy burden. Genotyping for HPV16/18 has been recommended in triaging HPV-positive women. This study was aimed to evaluate the screening performance of HPV testing and the role of genotyping triage in Northern Thailand.

**Methods:**

A population-based cervical screening program was performed in Chiang Mai (Northern Thailand) using cytology (conventional Pap test) and HPV test (Hybrid Capture 2). Women who had abnormal cytology or were HPV-positive were referred for colposcopy. Cervical samples from these women were genotyped using the Linear Array assay.

**Results:**

Of 5,456 women, 2.0% had abnormal Pap test results and 6.5% tested positive with Hybrid Capture 2. Of 5,433 women eligible for analysis, 355 with any positive test had histologic confirmation and 57 of these had histologic HSIL+. The sensitivity for histologic HSIL+ detection was 64.9% for Pap test and 100% for Hybrid Capture 2, but the ratio of colposcopy per detection of each HSIL+ was more than two-fold higher with Hybrid Capture 2 than Pap test (5.9 versus 2.8). Genotyping results were available in 316 samples. HPV52, HPV16, and HPV58 were the three most common genotypes among women with histologic HSIL+. Performance of genotyping triage using HPV16/18/52/58 was superior to that of HPV16/18, with a higher sensitivity (85.7% versus 28.6%) and negative predictive value (94.2% versus 83.9%).

**Conclusions:**

In Northern Thailand, HPV testing with genotyping triage shows better screening performance than cervical cytology alone. In this region, the addition of genotyping for HPV52/58 to HPV16/18 is deemed necessary in triaging women with positive HPV test.

## Introduction

Testing for high-risk HPV DNA (HPV test) has gained increasing acceptance as an alternative method to cervical cytology in primary cervical cancer screening [1]. It has been demonstrated HPV test has a higher sensitivity in the detection of cervical precancerous lesions than cytology, and women with negative HPV testing also have a lower cumulative incidence of cervical cancer and precancerous lesions compared to those with negative cytology [1–4]. HPV testing is a more objective method compared to cytological interpretation. While effective cytology screening requires a well-organized program with a good quality control, clinically validated HPV testing is automated and provides a more uniform performance over different geographic regions [4, 5]. In 2014, a high-risk HPV DNA test has been approved by the US FDA for use as primary cervical cancer screening for women 25 years or older, and, recently, interim guidance for the use of primary HPV screening has been published following this approval [1].

With the use of primary HPV screening, the number of women detected positive is higher than that of cytology screening [4]. Referral of all HPV-positive women for colposcopy would result in a large burden for gynecologists and triaging these patients to identify those with significant risk is needed. Triaging also help to decrease the expense and stress for the patients related to colposcopy [6]. Based on the guidance for primary HPV screening, HPV-positive women with genotypes HPV16/18 should be referred for immediate colposcopy, whereas those with other high-risk HPV genotypes should be triaged by cytology [1]. Women with abnormal cytology are referred for colposcopy, while those with negative cytology can be followed up after 12 months [1].

Geographic variation in the prevalence and oncogenic potential of HPV genotypes [7] may affect the performance of this triage approach. In Eastern Asia, HPV52 and HPV58 are more common among cervical cancers and precancerous lesions than in the other regions of the world [7–9]. In addition, studies that analyzed the sublineages or variants of HPV52 and HPV58 suggest that there are differences in variant distribution of these HPV genotypes across different geographic regions [10, 11]. Furthermore, the oncogenic potential of HPV52 and HPV58 may also vary with their variants, with a possible higher potential for the variants that are more prevalent in Eastern Asia [10–12]. Previous studies in Thailand have demonstrated that HPV58 and HPV52 were the most common genotypes in cervical cancer after HPV16 and HPV18 [13].

Thailand has a rather high incidence of cervical cancer with an age-standardized rate of 19.8 per 100,000 women, and Northern Thailand is among the most prevalent areas in the country [14]. Cytology screening using conventional Papanicolaou smears (Pap test) is the major screening method, and contributes to some recent decline in cervical cancer incidence. However, a well-organized cytology screening program has not yet been established in Thailand [14]. To facilitate the implementation of a primary HPV screening in Northern Thailand, several questions remain to be addressed. It is unclear how to manage the HPV-positive women, whether the genotyping triage for HPV16/18 could have a similar performance compared to the Western studies, and whether other genotypes (e.g. HPV52/58) which are common in this region have a role in triaging HPV-positive women. This study was aimed to evaluate the performance of HPV testing and the role of HPV genotyping in a population-based cervical cancer screening in Northern Thailand.

## Methods

### Study population

This cross-sectional study was approved by the institutional Ethics Committee of the Faculty of Medicine, Chiang Mai University (study code: PAT-11-02-07A-14-X). The study population included women aged 25 years or older who were residents in 3 prefectures (Sankumpang, Mae-on, Sarapee) of Chiang Mai, in Northern Thailand. These women participated in a cervical cancer screening scheduled by the Ministry of Health between May and December 2013. Women who were pregnant, had a previous hysterectomy, or had a previous history of any cervical epithelial lesion or positive cytology were excluded. Written informed consent was obtained from each participant.

### Sample collection

Cervical specimens were collected for cytology and HPV testing by trained nurses in local clinics for screening. From each woman, the first sample was taken for cytology using an Ayre spatula and was used to prepare a conventional Pap smear. The second sample was taken for HPV testing using the Digene Cervical Sampler (HC2 DNA Collection Device: Qiagen, Gaithersburg, MD). The cell sample was collected by a cytobrush and deposited into a tube with transport medium. The samples were stored in cool room and sent weekly for analysis to the Department of Pathology, Chiang Mai University.

### Cytology

The slides were screened by one of 2 cytotechnologists. When abnormal cells were detected, the slides were rescreened by at least one pathologist. All cytological interpretations were made blinded to the results of HPV testing. Abnormal or positive cytology was defined as atypical squamous cells of undetermined significance (ASC-US) or worse.

### HPV testing

Hybrid Capture 2 (HC2: Qiagen, Gaithersburg, MD) was used to detect high-risk HPV DNA. HC2 test is designed to detect 13 high-risk HPV genotypes (HPV16, 18, 31, 33, 35, 39, 45, 51, 52, 56, 58, 59, and 68; specific genotypes are not included in the result). Positive HC2 was defined using a cut-off ratio of relative light unit/ positive control ≥1.0, according to the manufacturer’s recommendation.

### Colposcopy

All women who had positive cytology or positive HC2 were referred for colposcopy with cervical biopsy, at the Colposcopy Clinic, Chiang Mai University Hospital. Colposcopy was performed by staffs or fellows in gynecologic oncology. If no lesion was seen colposcopically, at least one random cervical biopsy was obtained at the 12 o’clock position of the cervix. Women with cervical lesions were managed as per the standard guidelines [15].

### HPV genotyping

Cervical cell samples were collected from women with positive screening test results (cytology or HC2) before the colposcopy procedure. Cervical cell samples were collected and transferred into PreservCyt solution (Hologic, Marlborough, MA, USA). DNA extraction was performed using a DNeasy Blood and Tissue kit (Qiagen, Hilden, Germany). The samples were screened by PCR amplification using primers MY09/MY11 located within the HPV L1 gene. The samples that were negative with primers MY09/MY11 were re-amplified using primers GP5+/GP6+. The presence of co-amplified 199-bp fragment of beta-globin gene served as an internal standard for DNA quality and quantity of samples. Only the samples that were PCR-positive were further processed for genotyping. HPV genotyping was performed using the Linear Array HPV Genotyping Test (Roche Molecular System, Inc., Branchburg, NJ, USA) according to the manufacturer’s protocol. The Linear Array assay is designed to simultaneously identify 37 HPV genotypes. Interpretation of the presence of HPV52 was made only in the absence of HPV33, HPV35, and HPV58 due to cross reactivity of the probe in this assay [16].

### Histology

The histologic diagnosis used for analysis was the worst lesion identified in the colposcopic biopsy or subsequent specimens, based on the WHO Classification [17]. Histologic high-grade squamous intraepithelial lesion (or cervical intraepithelial neoplasia [CIN] 2 and 3) or worse lesions (HSIL+) were targets of detection. The histologic diagnosis of HSIL+ was made by a consensus of at least 3 pathologists and was based on the standard histomorphologic criteria [17, 18]. Immunohistochemical stain for p16 was used only in selected cases when a consensus was not reached.

### Data analysis

Correlations were made between histologic diagnosis, patient age, and the results of cytology, HC2, and HPV genotyping. The data were analyzed using STATA version 11 (StataCorp LP, College Station, TX, USA). Differences of the results were tested using Fisher Exact test. Accuracy values of triage methods in the prediction of histologic HSIL+ were calculated. Comparison of performance or accuracy of tests was assessed using a 2-sample proportion test (Z-test) and receiver operating characteristic (ROC) curve analysis. Diagnostic odds ratios (dOR) of the test methods were estimated by logistic regression analysis. A p value <0.05 was considered statistically significant.

## Results

### Characteristics of study population

**Fig 1:**
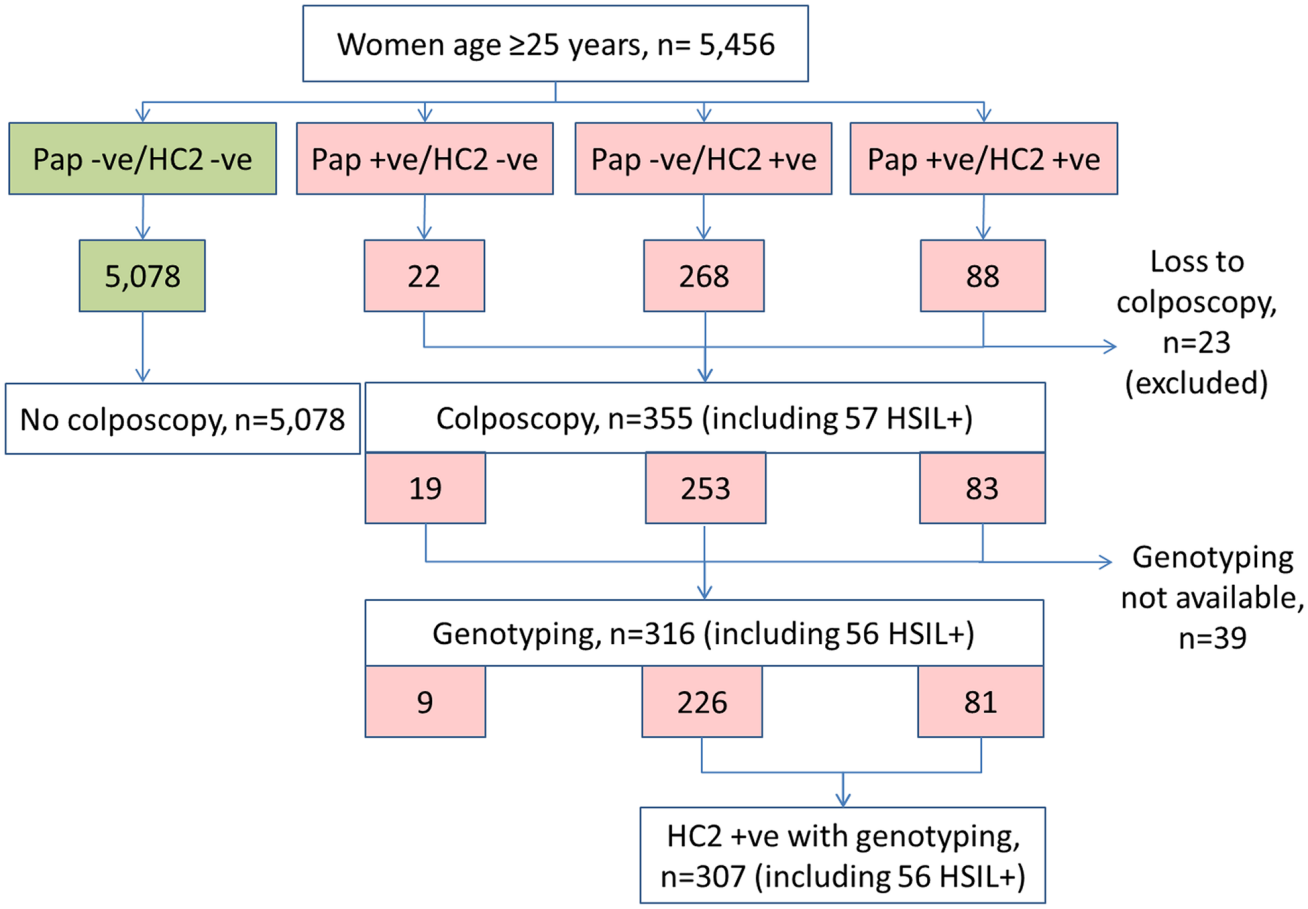
Flowchart for the study population. Pap, Pap test; HC2, Hybrid Capture 2; HSIL+, histologic high-grade squamous intraepithelial lesion or worse lesions.

There were 5,456 women attending for the screening, 5,078 of which (93.1%) had negative tests and 378 (6.9%) had either positive cytology or HC2 (Fig 1). Cytology was positive in 110 women (2.0%) and HC2 was positive in 356 women (6.5%), with 88 women positive for both tests (1.6%). Twenty-three women with positive test(s) were lost to colposcopy and were excluded. The mean age of the remaining 5,433 women (5,078 with negative tests and 355 with positive cytology and/or HC2) was 48.3±SD 7.8 years (range 26 to 65 years, median 50 years).

### Histology and genotyping results

Cervical histology was available in 355 women; 216 (60.8%) had negative histology, 82 (23.1%) had LSIL, and 57 (16.1%) had HSIL+ which included 10 women with squamous cell carcinoma. Women with histologic HSIL+ accounted for 1.0% of the study population. The rate of histologic HSIL+ did not significantly differ by age group using the cut-off of 35 years (1.0% vs 1.1%, p>0.99) or 45 years (1.4% vs 0.9%, p = 0.182). Genotyping results were available in 316 of these 355 women (excluding absence of samples in 5 women and negative-PCR samples in 34 women). Of these 316 women, 307 were HC2-positive and 56 had histologic HSIL+ (Fig 1). One of the 57 women with histologic HSIL+ had no genotype result due to missing sample collection.

Within the 316 samples from women with available histology and genotyping results (Table 1), the 13 high-risk HPV genotypes were identified in 263 samples (83.2%), whereas 37 samples had other HPV genotypes and the remaining 16 samples had undetermined genotype. All 56 women with histologic HSIL+ in this group had infections of at least one of 13 high-risk genotypes. The distribution of 13 high-risk HPV genotypes among the 263 samples is presented in Table 2. Multiple infections were detected in 8 of 56 (14.3%) women with histologic HSIL+, 25 of 79 (31.6%) women with histologic LSIL, and 31 of 181 (17.1%) women with negative histology. The rate of multiple infections in the histologic HSIL+ group was significantly lower than that of the histologic LSIL group (p = 0.025).

**Table 1:** Histologic diagnoses in 316 women with available histology and genotyping results.

HPV genotype^a^	Histology results, n = 316		
	Negative, n = 181	LSIL, n = 79	HSIL+, n = 56
**16, n = 42 (%)**	21 (50.0)	7 (16.7)	14 (33.3)
**18, n = 18 (%)**	11 (61.1)	5 (27.8)	2 (11.1)
**31, n = 21 (%)**	9 (42.9)	7 (33.3)	5 (23.8)
**33, n = 4 (%)**	3 (75.0)	0	1 (25.0)
**35, n = 4 (%)**	3 (75.0)	1 (25.0)	0
**39, n = 45 (%)**	27 (60.0)	15 (33.3)	3 (6.7)
**45, n = 4 (%)**	2 (50.0)	2 (50.0)	0
**51, n = 25 (%)**	13 (52.0)	11 (44.0)	1 (4.0)
**52, n = 102 (%)**	47 (46.1)	30 (29.4)	25 (24.5)
**56, n = 22 (%)**	14 (63.6)	7 (31.8)	1 (4.5)
**58, n = 18 (%)**	6 (33.3)	4 (22.2)	8 (44.4)
**59, n = 9 (%)**	6 (66.7)	2 (22.2)	1 (11.1)
**68, n = 29 (%)**	14 (48.3)	11 (37.9)	4 (13.8)
**Non-13 high-risk** ^b^, **n = 53 (%)**	45 (84.9)	8 (15.1)	0

HSIL+, histologic high-grade squamous intraepithelial lesion or worse lesions; LSIL, histologic low-grade squamous intraepithelial lesion

^a^ including single and multiple infections

^b^ exclusion of women with any infection of 13 high-risk genotypes

**Table 2:** Genotype distribution in 263 women with infections of 13 high-risk HPV genotypes.

HPV genotype	Single or multiple infections^a^		Single infection		Percentage of HSIL+ within each genotype
	No. (% of n = 263)	No. of HSIL+ (% of n = 56)	No. (% of n = 199)	No. of HSIL+ (% of n = 48)	
**16**	42 (16.0)	14 (25.0)	28 (14.1)	12 (25.0)	33.3
**18**	18 (6.8)	2 (3.6)	11 (5.5)	1 (2.0)	11.1
**31**	21 (8.0)	5 (8.9)	10 (5.0)	3 (6.3)	23.8
**33**	4 (1.5)	1 (1.8)	2 (1.0)	0 (0)	25.0
**35**	4 (1.5)	0 (0)	3 (1.5)	0 (0)	0
**39**	45 (17.1)	3 (5.4)	12 (6.0)	0 (0)	6.7
**45**	4 (1.5)	0 (0)	2 (1.0)	0 (0)	0
**51**	25 (9.5)	1 (1.8)	15 (7.5)	0 (0)	4.0
**52**	102 (38.8)	25 (44.6)	83 (41.7)	22 (45.8)	24.5
**56**	22 (8.4)	1 (1.8)	15 (7.5)	1 (2.1)	4.5
**58**	18 (6.8)	8 (14.3)	13 (6.5)	8 (16.7)	44.4
**59**	9 (3.4)	1 (1.8)	4 (2.0)	0 (0)	11.1
**68**	29 (11.0)	4 (7.1)	2 (1.0)	1 (2.1)	13.8

HSIL+, histologic high-grade squamous intraepithelial lesion or worse lesions

^a^ single or multiple infections of 13 high-risk genotypes

### Genotyping results and the detection of histologic HSIL+

HPV52, HPV16, HPV58, and HPV31 were the most common genotypes within 56 women with histologic HSIL+, either in the single infection group or combined single and multiple infections (Table 2). The percentage of detection of histologic HSIL+ was higher than 20% for HPV58 (44.4%), HPV16 (33.3%), HPV52 (24.5%), HPV33 (25.0%), and HPV31 (23.8%), but HPV33 were detected in only a few samples. HPV16, HPV18, HPV31, HPV52, and HPV58 were detected in 51 out of 56 (91.1%) women with histologic HSIL+.

Among 90 women with positive cytology and genotyping, the majority of women with histologic HSIL+ (91.7%) had HPV16, HPV18, HPV31, HPV52, and HPV58 detected. The rate of histologic HSIL+ in each of these genotypes, except HPV18, was 50% or above (HPV16, 55.6%; HPV31, 50.0%; HPV52, 50.0%; and HPV58, 100%). The presence of HPV16/18/52/58 or HPV16/18/31/52/58 was associated with a significantly higher risk for histologic HSIL+ than the absence of these genotypes (S1 Table). Using 8 genotypes (HPV16/18/31/33/35/45/52/58) for stratification [19], the detection of histologic HSIL+ was not improved compared to HPV16/18/31/52/58. A similar finding was observed among 226 women with positive HC2 but negative cytology (S2 Table). In this group, the rate of histologic HSIL+ in each genotype was above 10% for HPV16 (16.7%), HPV31 (13.3%), HPV52 (14.9%), and HPV58 (16.7%). Among all 316 women with known HPV genotypes, the odds ratios for histologic HSIL+ were 2.1 (95% confidence interval [CI]: 1.0–4.2) in women with HPV16/18, 6.9 (95% CI: 3.1–17.5) in those with HPV16/18/52/58, and 9.7 (95% CI: 3.7–32.1) in those with HPV16/18/31/52/58.

### Comparison of screening performance in the detection of histologic HSIL+

Table 3 shows a comparison of the different approaches of screening and triage methods in the detection of histologic HSIL+ and the estimated ratio of colposcopy per detection of each histologic HSIL+. The ratio of colposcopy per detection of histologic HSIL+ was the highest using positive cytology and/or positive HC2 (comparable to positive co-testing). Screening using HC2 alone resulted in a slightly lower ratio. Triaging of HC2-positive women by cytology resulted in the lowest ratio of colposcopy per HSIL+ detection, but 35.1% of women with histologic HSIL+ were missed. Genotyping triage of HC2-positive women using different groups of genotypes showed rather comparable ratios of colposcopy per HSIL+ detection, but the number of HSIL+ cases detected was lowest using genotypes HPV16/18. The addition of genotypes HPV52/58 or HPV31/52/58 to HPV16/18 apparently increased the number of HSIL+ detection without an increase of colposcopy per HSIL+ detection ratio. In the strategy of triaging HC2-positive women using HPV genotyping combined with cytology, the addition of genotypes HPV52/58 or HPV31/52/58 to HPV16/18 increased the proportion of HSIL+ detection from 71.9% to 94.7–96.5%, but the colposcopy per HSIL+ detection ratios were also increased from 2.8 to 3.6–3.7. There were 2 cases of histologic HSIL+ that were missed by combined HPV 16/18/31/52/58 genotyping and cytology triage (Table 3); one was a 57-year-old woman who had co-infection of HPV39 and HPV68, and the other was a 59-year-old woman who had HPV68 infection. Both women had histologic HSIL identified in colposcopy-directed biopsy specimens, whereas their conization specimens showed no residual epithelial lesion.

**Table 3:** Detection of histologic high-grade squamous intraepithelial lesion or worse lesions by different screening approaches in the study population.

Screening approach	No. of positive tests	No. of HSIL+ detections	Percentage of HSIL+ detected, n = 57^a^	Percentage of HSIL+ among cases with positive test	Ratio of colposcopy per HSIL+ detection^b^
**Positive cytology and/or positive HC2**	355	57	100	16.1	6.2
**Positive HC2**	336	57	100	17.0	5.9
**Positive cytology**	102	37	64.9	36.3	2.8
**Positive HC2 and triage by cytology**	83	37	64.9	44.6	2.2
**Positive HC2 and triage by genotyping**					
HPV 16/18	58	16	28.1	27.6	3.6
HPV 16/18/52/58	169	48	84.2	28.4	3.5
HPV 16/18/31/52/58	184	51	89.5	27.7	3.6
HPV 8 genotypes^c^	191	51	89.5	26.7	3.7
**Positive HC2 and triage by combined genotyping and cytology**					
HPV 16/18	116^d^	41	71.9	35.3	2.8
HPV 16/18/52/58	194^d^	54	94.7	27.8	3.6
HPV 16/18/31/52/58	205^d^	55	96.5	26.7	3.7
HPV 8 genotypes^c^	211^d^	55	96.5	26.1	3.8

HSIL+, histologic high-grade squamous intraepithelial lesion or worse lesions

^a^ equivalent to crude sensitivity in the detection of histologic HSIL+

^b^ representing a ratio between the number of positive tests to the number of histologic HSIL+ detections

^c^ 8 genotypes including HPV16/18/31/33/35/45/52/58

^d^ including 2 women with positive cytology but without genotyping results

### Comparison of performance of triage methods for HC2-positive women

Considering genotyping triage strategy for HC2-positive women, the performance of different triage methods was evaluated in the group of 307 women with genotyping results. The accuracy values in the prediction of histologic HSIL+ are shown in Table 4. Cytology had a low sensitivity (64.3%) but the specificity was high (82.1%). Genotyping for HPV16/18 had the lowest sensitivity but had a high specificity comparable to that of cytology. The sensitivity of HPV16/18 genotyping was significantly lower than that of cytology (p<0.001), whereas genotyping for at least HPV16/18/52/58 had a significantly higher sensitivity than cytology (p<0.001). Genotyping for at least HPV16/18/52/58 also showed a higher negative predictive value (NPV) than HPV16/18. The use of 8 HPV genotypes did not improve the performance of HPV16/18/31/52/58. Combination of cytology with HPV genotyping increased the sensitivity and negative predictive value when compared with genotyping triage alone. In the subgroups of women <45 years and women ≥45 years, similar findings were observed (Table 4).

**Table 4:** Triaging 307 women with positive Hybrid Capture 2 and available genotyping results using different methods.

Triage method	No. of HSIL+	Sensitivity	Specificity	PPV	NPV
**Overall group, n = 307**	56				
**Positive cytology, n = 81**	36	64.3 (50.4–76.6)	82.1 (76.8–86.6)	44.4 (33.4–55.9)	91.2 (86.7–94.5)
**Genotyping**					
HPV16/18, n = 58	16	28.6 (17.3–42.2)	83.3 (78.1–87.7)	27.6 (16.7–40.9)	83.9 (78.8–88.3)
HPV16/18/52/58, n = 169	48	85.7 (73.8–93.6)	51.8 (45.4–58.1)	28.9 (21.7–35.8)	94.2 (88.9–97.5)
HPV16/18/31/52/58, n = 184	51	91.1 (80.4–97.0)	47.0 (40.7–53.4)	27.7 (21.4–34.8)	95.9 (90.8–98.7)
HPV 8 genotypes^a^, n = 191	51	91.1 (80.4–97.0)	44.2 (38.0–50.6)	26.7 (20.6–33.6)	95.9 (90.2–98.6)
**Combined genotyping and cytology**					
HPV16/18, n = 114	40	71.4 (57.8–82.7)	70.5 (64.5–76.1)	35.1 (26.4–44.6)	91.7 (86.9–95.2)
HPV16/18/52/58, n = 192	53	94.6 (85.1–98.9)	44.6 (38.4–51.0)	27.6 (21.4–34.5)	97.4 (92.6–99.5)
HPV16/18/31/52/58, n = 203	54	96.4 (87.7–99.6)	40.6 (34.5–47.0)	26.6 (20.7–33.2)	98.1 (93.2–99.8)
HPV 8 genotypes^a^, n = 209	54	96.4 (87.7–99.6)	38.2 (32.2–44.6)	25.8 (20.0–32.3)	98.0 (92.8–99.8)
**Age group <45 years, n = 112**	21				
**Positive cytology, n = 34**	13	61.9 (38.4–81.9)	76.9 (66.9–85.1)	38.2 (22.2–56.4)	89.7 (80.8–95.5)
**Genotyping**					
HPV16/18, n = 23	7	33.3 (14.6–57.0)	82.4 (73.0–89.6)	30.4 (13.2–52.9)	84.3 (75.0–91.1)
HPV16/18/52/58, n = 60	19	90.5 (69.6–98.8)	54.9 (44.2–65.4)	31.7 (20.3–45.0)	96.2 (86.8–99.5)
HPV16/18/31/52/58, n = 68	20	95.2 (76.2–99.9)	47.3 (36.7–58.0)	29.4 (19.0–41.7)	97.7 (88.0–99.9)
HPV 8 genotypes^a^, n = 70	20	95.2 (76.2–99.9)	45.1 (34.6–55.8)	28.6 (18.4–40.6)	97.6 (87.4–99.9)
**Combined genotyping and cytology**					
HPV16/18, n = 47	15	71.4 (47.8–88.7)	64.8 (54.1–74.6)	31.9 (19.1–47.1)	90.8 (81.0–96.5)
HPV16/18/52/58, n = 71	21	100 (83.9–100.0)	45.1 (34.6–55.8)	29.6 (19.3–41.6)	100 (91.4–100.0)
HPV16/18/31/52/58, n = 77	21	100 (83.9–100.0)	38.5 (28.4–49.2)	27.3 (17.7–38.6)	100 (90.0–100.0)
HPV 8 genotypes^a^, n = 78	21	100 (83.9–100.0)	37.4 (27.4–48.1)	26.9 (17.5–38.2)	100 (89.7–100.0)
**Age group ≥45 years, n = 195**	35				
**Positive cytology, n = 47**	23	65.7 (47.8–80.9)	85.0 (78.5–90.1)	48.9 (34.1–63.9)	91.9 (86.3–95.7)
**Genotyping**					
HPV16/18, n = 35	9	25.7 (12.5–43.3)	83.8 (77.1–89.1)	25.7 (12.5–43.3)	83.8 (77.1–89.1)
HPV16/18/52/58, n = 109	29	82.9 (66.4–93.4)	50.0 (42.0–58.0)	26.6 (18.6–35.9)	93.0 (85.4–97.4)
HPV16/18/31/52/58, n = 116	31	88.6 (73.3–96.8)	46.9 (39.0–54.9)	26.7 (18.9–35.7)	94.9 (87.5–98.6)
HPV 8 genotypes^a^, n = 121	31	88.6 (73.3–96.8)	43.8 (35.9–51.8)	25.6 (18.1–34.4)	94.6 (86.7–98.5)
**Combined genotyping and cytology**					
HPV16/18, n = 67	25	71.4 (53.7–85.4)	73.8 (66.2–80.4)	37.3 (25.8–50.0)	92.2 (86.1–96.2)
HPV16/18/52/58, n = 121	32	91.4 (76.9–98.2)	44.4 (36.5–52.4)	26.4 (18.8–35.2)	95.9 (88.6–99.2)
HPV16/18/31/52/58, n = 126	33	94.3 (80.8–99.3)	41.9 (34.1–49.9)	26.2 (18.8–34.8)	97.1 (89.9–99.6)
HPV 8 genotypes^a^, n = 131	33	94.3 (80.8–99.3)	38.8 (31.2–46.8)	25.2 (18.0–33.5)	96.9 (89.2–99.6)

HSIL+, histologic high-grade squamous intraepithelial lesion or worse lesions; NPV, negative predictive value; PPV, positive predictive value

^a^ 8 genotypes including HPV16/18/31/33/35/45/52/58

**Fig 2:**
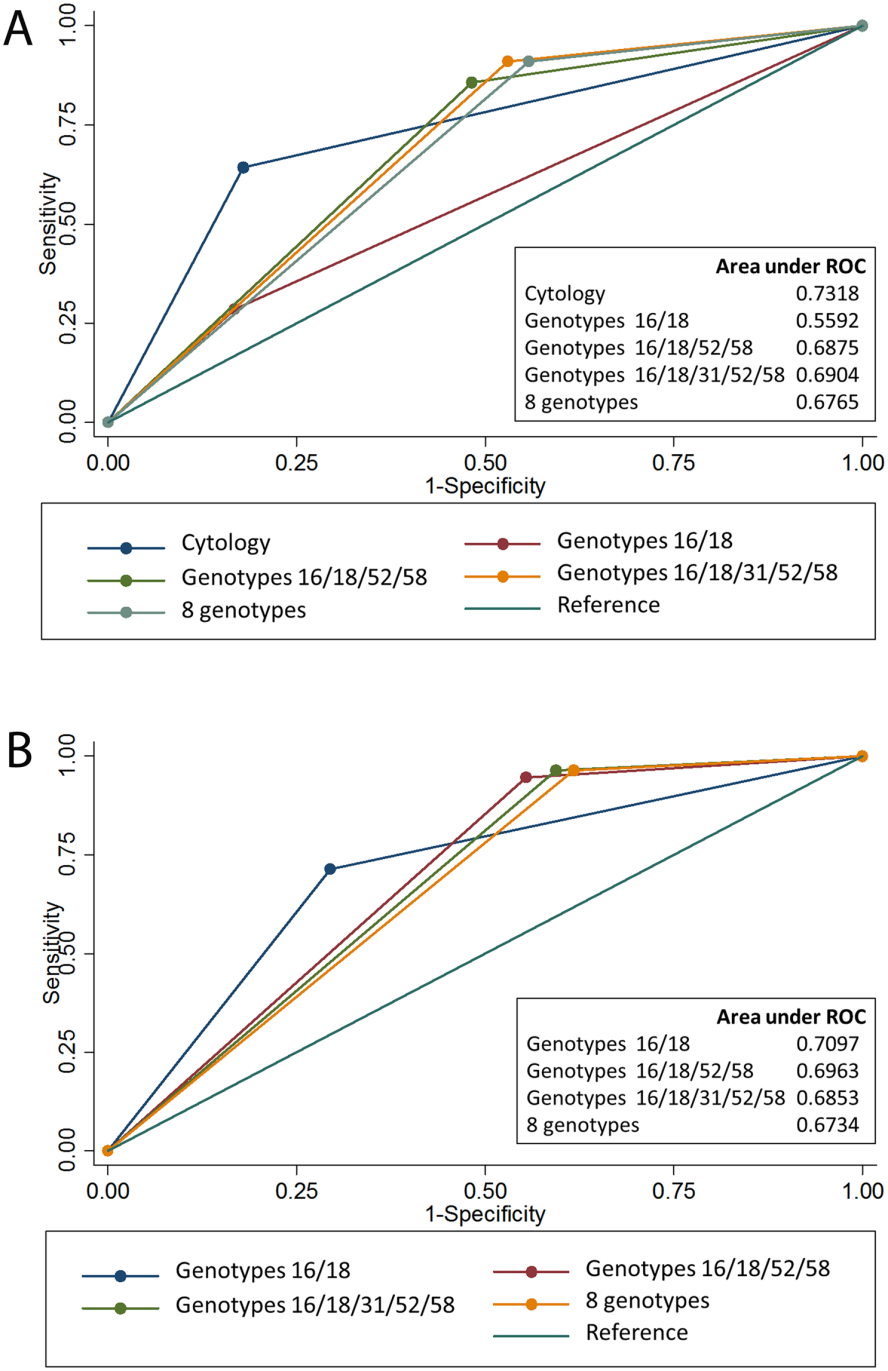
Receiver operating curve analysis comparing triage methods in 307 women with positive Hybrid Capture 2. (A) triage using cytology or genotyping (p<0.001). (B) triage using combined genotyping and cytology (p = 0.055).

Receiver operating curve (ROC) analysis of different triage methods for HC2-positive women is shown in Fig 2. The area under ROC of HPV16/18 triage was lower than cytology triage or other genotyping triages (Fig 2A). In the combined strategies using genotypes and cytology, the differences of area under ROC between the four methods of genotyping did not reach statistical significance (Fig 2B).

In a comparison of triage performance by dOR, combined genotyping for HPV16/18/31/52/58 and cytology showed the highest value (Table 5). The dOR of triage method using HPV16/18/52/58 genotyping alone was slightly higher than that of combined HPV16/18 genotyping and cytology (6.4 vs 5.9). Triage methods that combined cytology to genotyping for at least HPV16/18/52/58 showed over a 2-fold diagnostic odds ratio compared to combined HPV16/18 genotyping and cytology.

**Table 5:** Comparison of diagnostic odds ratios of triage methods in 307 women with positive Hybrid Capture 2 and HPV genotyping.

Triage method	Diagnostic odds ratios (95% CI)	p-value
**Cytology**	8.2 (4.4–15.5)	<0.001
**Genotyping**		
HPV16/18	1.9 (1.0–3.9)	0.043
HPV16/18/52/58	6.4 (2.9–14.2)	<0.001
HPV16/18/31/52/58	9.0 (3.5–23.4)	<0.001
HPV 8 genotypes^a^	8.1 (3.1–20.9)	<0.001
**Combined genotyping and cytology**		
HPV16/18	5.9 (3.2–11.3)	<0.001
HPV16/18/52/58	14.2 (4.3–46.8)	<0.001
HPV16/18/31/52/58	18.5 (4.4–77.5)	<0.001
HPV 8 genotypes^a^	16.7 (3.9–70.2)	<0.001

^a^ 8 genotypes including HPV16/18/31/33/35/45/52/58

## Discussion

This study shows that the triage method for HPV-positive women using HPV16/18 genotyping may have a lower performance in the detection of histologic HSIL+ in Northern Thailand. It also shows that additional genotyping for at least HPV52/58 may help to improve the triage performance in this region. Similar to the findings in previous studies, HPV screening had a superior sensitivity for the detection of histologic HSIL+ compared with screening by Pap test.

Cytology screening in this study represents a real-world practice in Thailand where conventional Pap test is used in cervical cancer screening. The cytology-positive rate in this study is rather low (2.0%), but it is slightly higher than the 1.6% overall prevalence of positive cytology in Thailand [14]. In a previous study in women from the same area [20], the positive cytology rate by liquid-based cytology screening was 4.3%, which suggests that the difference may be related to the use of conventional Pap test. Although liquid-based cytology may be more sensitive than conventional Pap test in the detection of cervical epithelial abnormalities, there was no significant difference in the detection of histologic HSIL+ between both methods in previous studies [21, 22]. The rate of positive Pap test and the rate of histologic HSIL+ in this study (2.0% and 1.0%, respectively) is comparable to the rates reported in a study with similar setting in Chile (1.7% and 1.1%, respectively) [23]. The crude sensitivity of Pap test for the detection of histologic HSIL+ in this study (64.9%, Table 3) was comparable to the sensitivity of Pap test reported from European countries (63.5%) [24].

The rate of positive HPV testing in this study (6.5%) is slightly lower than that reported in a previous study from the same region (7.1%) [20], but is comparable to the rate of high-risk HPV infection in a recent study in central Thailand which used the Linear Array Genotyping Test (6.4%) [25]. The rather low HPV-positive rate in Thailand makes the implementation of primary HPV screening practical because of the number of patients who require further triaging and colposcopy is not overwhelming [25], compared to some other countries where an almost 20% HPV-positive rate has been reported [26, 27]. As a cytology screening program has not been well-organized in Thailand [14], HPV testing may offer a more uniform screening performance. The guidance for management approach of women with positive HPV testing was also rather simple to follow [1]. It is noteworthy that cytology still has an important role in triaging women with positive HPV testing as suggested in the guidance, but the use of primary HPV screening can help to reduce a large amount of cytology workload in this area of limited resources/personnel. The major disadvantage of HPV testing is its currently high cost; however, it may be possible that the cost can be reduced with future technical advances and the larger volume of use.

In contrast to the other regions of the world, HPV52 and HPV58 are the most common genotypes found in cervical cancers after HPV16 and HPV18 in Eastern Asia and in Thailand [7, 9, 13, 28, 29]. The prevalence of HPV52 and HPV58 among HSIL and cervical cancer in Eastern Asia is almost 2-fold higher than that of the other regions [7]. In this study, HPV52 was the most common genotype in women with positive screening tests, similar to the results of previous studies in Eastern Asia and Thailand [25]. This finding is different from the reported data from the Western studies [25, 30]. Compared to a large US study [30], the risk of histologic HSIL+ among HPV16-positive women in the present study (33.3% among combined single and multiple infections) was higher than that previously reported (19.5% by hierarchical ranking calculation), but the risk for histologic HSIL+ in women testing positive for HPV52 and HPV58 in this study was even higher (24.5% vs 7.9% for HPV52, and 44.4% vs 6.1% for HPV58) [30]. The higher risk for histologic HSIL+ related to HPV52 and HPV58 in this study was also observed in the positive cytology subgroup (50% vs 13.5% for HPV52, and 100% vs 18.4% for HPV58) and negative cytology subgroup (14.9% vs 6.5% for HPV52, and 16.7% vs 3.9% for HPV58). Using HPV16 as a reference, the ratio of risk for histologic HSIL+ of HPV52 and HPV58 relative to HPV16 was higher in this study than in the US study (0.7 vs 0.4 for HPV52, and 1.3 vs 0.3 for HPV58) [30]. In the present study, HPV58-positive women had a higher rate of histologic HSIL+ than HPV16-positive women (44.4% vs 33.3%). This correlates with the finding in a Korean study that cytology-negative women with HPV58 infection were found to have a higher risk of developing histologic HSIL on follow-ups than those with HPV16 [31]. HPV33 and HPV45 have been found to have an important oncogenic risk [7, 32], but the prevalence of HPV33 and HPV45 was low in this study and none of single infection of these genotypes had histologic HSIL+.

In previous studies, HPV testing with HPV16/18 genotyping triage showed at least similar performance in the detection of histologic HSIL+ compared to cytology screening, with reported sensitivity ranging from 51.8% to 58.5% [33, 34]. When HPV16/18 genotyping was combined with cytology in triaging, the sensitivity was increased to 74.5–92.6% [33, 34]. In the present study, triaging HPV-positive women using HPV16/18 genotyping showed a lower sensitivity than previously reported, and the addition of at least HPV52/58 genotyping is deemed necessary. The addition of genotypes HPV52/58 or HPV31/52/58 to HPV16/18 genotyping triage improved the stratification of HPV-positive women who were at risk of histologic HSIL+ in both cytology-positive and cytology-negative subgroups. Compared to the triage using HPV16/18 genotyping, additional HPV52/58 genotyping triage increased the detection of patients with histologic HSIL+ from 28.1% to over 84% (Table 3), whilst the ratio of colposcopy per detection of HSIL+ was not increased. Similarly, when cytology was combined with genotyping triage, the proportion of HSIL+ detection increased from 71.9% (for HPV16/18) to over 94% (for HPV16/18/52/58). With an addition of HPV52/58 in triaging (Table 3), the need for cytology in triaging HPV-positive women could be reduced by 40% (from 278 women of non-HPV16/18 group to 167 women of non-HPV16/18/52/58 group).

Addition of HPV52/58 to HPV16/18 in triaging HPV-positive women (Table 4) improved the sensitivity by almost 3 folds (28.6% to over 85%) with a higher NPV, although the specificity dropped from 83.3% to 51.8%. The performance of genotyping triage was similar in the different age groups (<45 years and ≥45 years). The area under ROC was higher for multiple genotypes (at least HPV16/18/52/58) compared to HPV16/18. Cytology alone had a slightly higher area under ROC than the triage methods using multiple genotypes; however, the sensitivity of the triage using multiple genotypes was over 20% higher than that of cytology triage. The improvement in triage performance using multiple genotypes compared to HPV16/18 triage was similarly observed when cytology was combined with genotyping in the triage of HPV-positive women.

There were several limitations in this study. The number of participants in this study was rather limited, and follow-up results after colposcopy or negative screening tests have not been available. Further studies are necessary to confirm the usefulness of additional HPV52/58 genotyping in the regions with a high prevalence of HPV52 and HPV58, particularly in Eastern Asia. In this study, there was also no control group for verification bias adjustment from women with negative tests; however, these women appear to have a very low risk of histologic HSIL+ [35]. The 3-year cumulative absolute risk of histologic HSIL+ among HC2-negative women was reported to be only 0.26% [4]. A study from Chile reported a 2.2% rate of histologic HSIL+ (5 of 230) in women with negative Pap and HPV testing who were selected by the presence of clinical risks of cervical lesion from a total of 7,334 women with negative screening tests [23].

Another limitation in this study is that HPV genotyping was not performed on the same specimen used for HPV testing due to insufficient residual volume of samples after HC2 testing. Although this factor may be a possible cause in some cases for the discordance between positive HC2 (detecting any of 13 high-risk genotypes) and the absence of high-risk genotypes by Linear Array assay, the agreement between HC2 and Linear Array assay for the detection of high-risk HPV genotypes in this study was 83.2% which was comparable to the reported rate of 83.8% in another study [36]. All 56 samples from women with histologic HSIL+ and available genotyping results in this study were HC2-positive and had at least one of 13 high-risk genotypes detected by the Linear Array assay.

## Conclusions

Primary HPV screening appears to be more sensitive than screening by conventional Pap test in Northern Thailand. Due to the geographic variation in HPV genotype distribution and possibly oncogenic potential of some genotypes, modification of genotyping triage strategy for HPV-positive women may improve the performance in the detection of histologic HSIL+ and reduce the use of cytology in triaging. Genotyping for at least HPV52/58 should be added to the previously recommended HPV16/18 genotyping in triaging HPV-positive women in this region.

## Supporting Information

S1 Table Genotyping results and histology in 90 women with positive cytology. (DOCX)

S2 Table Genotyping results and histology in 226 women with positive Hybrid Capture 2 and normal cytology. (DOCX)
