# Supplementary material for: Genotyping for Human Papillomavirus (HPV) 16/18/52/58 Has a Higher Performance than HPV16/18 Genotyping in Triaging Women with Positive High-risk HPV Test in Northern Thailand
Source: PLoS One. 2016 Jun 23;11(6):e0158184. doi: 10.1371/journal.pone.0158184 (PMC4918932; doi:10.1371/journal.pone.0158184)
Supplement: S1 Table — (DOCX) [file pone.0158184.s001.docx]

**S1 Table. Genotyping results and histology in 90 women with positive cytology.**

| **HPV genotype(s)^a^** | **No. (% of n=90)** | **No. of HSIL+ (% of n=36)** | **Percentage of HSIL+ within genotype(s)** |
| --- | --- | --- | --- |
| **16** | 18 (20.0) | 10 (27.8) | 55.6 |
| **18** | 8 (8.9) | 2 (5.6) | 25.0 |
| **31** | 6 (6.7) | 3 (8.3) | 50.0 |
| **33** | 2 (2.2) | 1 (2.8) | 50.0 |
| **35** | 0 (0) | 0 (0) | 0 |
| **39** | 7 (7.8) | 2 (5.6) | 28.6 |
| **45** | 1 (1.1) | 0 (0) | 0 |
| **51** | 8 (8.9) | 1 (2.8) | 12.5 |
| **52** | 28 (31.1) | 14 (38.9) | 50.0 |
| **56** | 5 (5.6) | 1 (2.8) | 20.0 |
| **58** | 6 (6.7) | 6 (16.7) | 100 |
| **59** | 1 (1.1) | 1 (2.8) | 100 |
| **68** | 4 (4.4) | 2 (5.6) | 50.0 |
|  |  |  |  |
| **16/18** | 25 (27.8) | 12 (33.3) | 48.0, p=0.349 |
| **Non-16/18** | 65 (72.2) | 24 (66.7) | 36.9 |
| **16/18/52/58** | 58 (64.4) | 31 (86.1) | 53.4, p<0.001 |
| **Non-16/18/52/58** | 32 (35.6) | 5 (13.9) | 15.6 |
| **16/18/31/52/58** | 62 (68.9) | 33 (91.7) | 53.2, p<0.001 |
| **Non-16/18/31/52/58** | 28 (31.1) | 3 (8.3) | 10.7 |
| **8 genotypes^b^** | 63 (70.0) | 33 (91.7) | 52.4, p<0.001 |
| **Non-8 genotypes^b^** | 27 (30.0) | 3 (8.3) | 11.1 |

HSIL+, histologic high-grade squamous intraepithelial lesion or worse lesions

^a^ including single or multiple HPV infections

^b^ 8 genotypes including HPV16/18/31/33/35/45/52/58
