# Supplementary material for: Genotyping for Human Papillomavirus (HPV) 16/18/52/58 Has a Higher Performance than HPV16/18 Genotyping in Triaging Women with Positive High-risk HPV Test in Northern Thailand
Source: PLoS One. 2016 Jun 23;11(6):e0158184. doi: 10.1371/journal.pone.0158184 (PMC4918932; doi:10.1371/journal.pone.0158184)
Supplement: S2 Table — (DOCX) [file pone.0158184.s002.docx]

**S2 Table. Genotyping results and histology in 226 women with positive Hybrid Capture 2 and normal cytology.**

| **HPV genotype(s)^a^** | **No. (% of n=226)** | **No. of HSIL+ (% of n=20)** | **Percentage of HSIL+ within genotype(s)** |
| --- | --- | --- | --- |
| **16** | 24 (10.6) | 4 (20.0) | 16.7 |
| **18** | 10 (4.4) | 0 (0) | 0 |
| **31** | 15 (6.6) | 2 (10.0) | 13.3 |
| **33** | 2 (0.9) | 0 (0) | 0 |
| **35** | 4 (1.8) | 0 (0) | 0 |
| **39** | 38 (16.8) | 1 (5.0) | 2.6 |
| **45** | 3 (1.3) | 0 (0) | 0 |
| **51** | 17 (7.5) | 0 (0) | 0 |
| **52** | 74 (32.7) | 11 (55.0) | 14.9 |
| **56** | 17 (7.5) | 0 (0) | 0 |
| **58** | 12 (5.3) | 2 (10.0) | 16.7 |
| **59** | 8 (3.5) | 0 (0) | 0 |
| **68** | 25 (11.1) | 2 (10.0) | 8.0 |
|  |  |  |  |
| **16/18** | 33 (14.6) | 4 (20.0) | 12.1, p=0.505 |
| **Non-16/18** | 193 (85.4) | 16 (80.0) | 8.3 |
| **16/18/52/58** | 111 (49.1) | 17 (85.0) | 15.3, p<0.001 |
| **Non-16/18/52/58** | 115 (50.9) | 3 (15.0) | 2.6 |
| **16/18/31/52/58** | 122 (54.0) | 18 (90.0) | 14.8, p<0.001 |
| **Non-16/18/31/52/58** | 104 (46.0) | 2 (10.0) | 1.9 |
| **8 genotypes^b^** | 128 (56.6) | 18 (90.0) | 14.1, p=0.003 |
| **Non-8 genotypes^b^** | 98 (43.4) | 2 (10.0) | 2.0 |

HSIL+, histologic high-grade squamous intraepithelial lesion or worse lesions

^a^ including single or multiple HPV infections

^b^ 8 genotypes including HPV16/18/31/33/35/45/52/58
